# Clinical Effects of Laser Acupuncture plus Chinese Cupping on the Pain and Plasma Cortisol Levels in Patients with Chronic Nonspecific Lower Back Pain: A Randomized Controlled Trial

**DOI:** 10.1155/2017/3140403

**Published:** 2017-08-07

**Authors:** Mu-Lien Lin, Jih-Huah Wu, Chi-Wan Lin, Chuan-Tsung Su, Hung-Chien Wu, Yong-Sheng Shih, I-Ting Chiu, Chao-Yi Chen, Wen-Dien Chang

**Affiliations:** ^1^Institute of Biomedical Engineering, National Taiwan University, Taipei, Taiwan; ^2^Department of Pain Management, Taipei City Hospital Zhongxing Branch, Taipei, Taiwan; ^3^Department of Biomedical Engineering, Ming Chuan University, Taoyuan, Taiwan; ^4^Yi Sheng Chinese Medicine Clinic, Taipei, Taiwan; ^5^Department of Sports Medicine, China Medical University, Taichung City, Taiwan

## Abstract

**Objectives:**

Chronic nonspecific lower back pain (LBP) is a common disease. Insufficient data is currently available to conclusively confirm the analgesic effects of laser acupuncture on LBP. This study evaluated the effectiveness of laser acupuncture plus Chinese cupping in LBP treatment.

**Methods:**

Patients with chronic nonspecific LBP were enrolled for a randomized controlled trial and assigned to the laser acupuncture group (laser acupuncture plus Chinese cupping) and control group (sham laser plus Chinese cupping). Laser acupuncture (808 nm; 40 mW; 20 Hz; 15 J/cm^2^) and Chinese cupping were applied on the Weizhong (BL40) and Ashi acupoints for 5 consecutive days. Plasma cortisol levels were assessed before and after the 5-day treatment session. The visual analog scale (VAS) scores were recorded at baseline and throughout the 5-day treatment session.

**Results:**

After the treatment session, the plasma cortisol levels and VAS scores decreased significantly in both groups. In the laser acupuncture group, the VAS scores decreased significantly on days 4 and 5, and an enhanced reduction in VAS scores was observed.

**Conclusion:**

Laser acupuncture plus Chinese cupping at the Weizhong (BL40) and Ashi acupoints effectively reduced pain and inflammation in chronic nonspecific LBP. This therapy could be a suitable option for LBP treatment in clinical settings.

## 1. Introduction 

Chronic nonspecific lower back pain (LBP) is commonly observed in people aged <45 years in the United States. Furthermore, LBP is the second most common reason for clinic visits and the fifth most common cause of admission to hospitals for surgical procedures [1]. LBP causes spinal instability and eventually becomes chronic symptom [2]. However, conservative treatments are the first choice for patients with chronic nonspecific LBP. Acupuncture is one of the most popular treatments applied in traditional Chinese medicine and has been used for relieving pain in musculoskeletal diseases [3]. Acupuncture has been adopted as a clinical practice guideline for LBP by the National Health Insurance System in Taiwan. A systematic review and meta-analysis revealed that acupuncture treatment for LBP has moderate effects on pain relief and results in decreased functional limitations at short-term follow-up [4]. Chinese cupping is another traditional Chinese medicine treatment and uses open side of a cup against the skin, causing air pumping to decrease the air pressure in the cup. It could cause local stasis of blood, qi activation, and muscle microcirculation [5]. Yuan et al. indicated that Chinese cupping had good effects on reducing muscle pain and revealed that it was more effective at decreasing pain than analgesic in LBP patients [6]. Combing acupuncture with Chinese cupping, the therapeutic effects can be enhanced [5]. Therefore, acupuncture plus Chinese cupping may be used as an immediate treatment for reducing LBP.

In traditional Chinese medicine treatment, the clinical value of acupuncture for musculoskeletal disease has been sufficiently established [7, 8]. Laser acupuncture is a type of low-level laser therapy (LLLT) on acupoints and is similar to needle acupuncture. Karu explored the biostimulation effect of LLLT on cells [9]. In vitro, adenosine triphosphate can be activated after He–Ne low-level laser irradiated on cells [10]. The photobiomodulation process involves light absorption by a photoreceptor and leads to signal transduction and molecular modulations in DNA and RNA syntheses caused by low-level laser radiation [11]. A systematic review and meta-analysis of in vivo studies revealed that low-level laser acupuncture progressively alleviates temporomandibular disorders, rheumatoid arthritis and gingival healing, and musculoskeletal pain [12]. Moderate evidence supports that laser acupuncture exerts positive effects on musculoskeletal diseases [12]. Furthermore, LLLT exerts additional effects on LBP, including the inhibition of central nerve synaptic activity, peripheral nerve blockade, neurotransmitter modulation, and muscle spasm reduction [13]. However, studies on laser acupuncture for LBP are rare. Lin et al. used laser acupuncture plus soft cupping on the Weizhong (BL40) acupoints for treating patients with LBP and demonstrated that laser acupuncture can increase the relative meridian values in Ryodoraku analysis and alleviate pain [14]. Although evidence from traditional Chinese medicine has provided an explanation for these effects, the analgesic effects of laser acupuncture are yet to be conclusively confirmed. Therefore, we administered laser acupuncture plus Chinese cupping to patients with chronic nonspecific LBP and recorded their plasma cortisol levels and visual analog scale (VAS) scores to assess the changes after the treatment.

## 2. Methods

### 2.1. Participant Selection

Patients from the Orthopedic Department of Taipei Municipal Chung-Hsin Hospital were included as study participants. Patients experiencing chronic nonspecific LBP for at least 3 months between the 12th rib and gluteal fold were included in this study. Patients who were pregnant and had other conditions, such as chronic obstructive pulmonary disease, were excluded. They were requested not to intake drug and use ointment containing steroid. Low dosage (500 mg) of acetaminophen (one time a day) was administered to the laser acupuncture and control groups during the study, if the pain was absolutely unbearable. Sample size was estimated using G^*∗*^Power software (G^*∗*^Power 3.1.9.2, Heinrich-Heine-Universität Düsseldorf, Germany) and by referring to the patient data reported by Lin et al. [14]. The means and standard deviations of the assessed VAS scores in the experimental and control groups of the previous study results were calculated, and the mean and standard deviation of the difference were 0.08 and 0.17, respectively. The estimated sample size (*α* = 0.05 and power = 0.80) in our study was 20–25 patients per group. Finally, 50 patients who received a diagnosis of chronic nonspecific LBP were recruited in our study. When we enrolled the participants, 2 patients, who had other physical conditions, were excluded because they did not satisfy the inclusion criteria. Randomization was conducted after 48 patients completed signing an informed consent form. The sequence generation process was based on a computer-generated random number table using Microsoft Excel (Microsoft Corp., Redmond, WA, USA). Blinding of patients was achieved using a random number, where odd digit in ones is assigned to laser acupuncture group (laser instrument number 1 plus Chinese cupping) and even digit in ones is assigned to control group (laser instrument number 2 plus Chinese cupping). There are 25 patients in laser acupuncture group and 23 patients in control group. This study was approved by the Ethical Committee of Taipei Municipal Chung-Hsin Hospital (number TCHIRB-990304).

### 2.2. Procedure

This study is a randomized controlled trial. The patients with chronic nonspecific LBP who were assigned to the laser acupuncture and control groups received laser acupuncture plus Chinese cupping and sham laser acupuncture plus Chinese cupping, respectively. All the procedures were done in Taipei Municipal Chung-Hsin Hospital. Real laser acupuncture was applied by the laser instrument (number 1), and sham laser acupuncture was applied by the laser instrument (number 2). Sham laser acupuncture was performed to appear like real laser instrument, applying the same treatment procedure but without energy output. All patients were blinded to receiving a true laser (number 1) or sham laser (number 2) acupuncture by a physician (Lin M. L.). Plasma cortisol levels were recorded before and after the treatment session. The VAS scores for LBP were recorded at baseline and during days 1–5 of the treatment session. The outcomes data were collected and analyzed by an assessor (Wu J. H.). The physician and assessors were blinded to laser instrument and group assignments until the data were analyzed. Allocation concealment was achieved using opaque sealed envelopes containing information of the assignments.

### 2.3. Treatment Protocol

**Figure 1:**
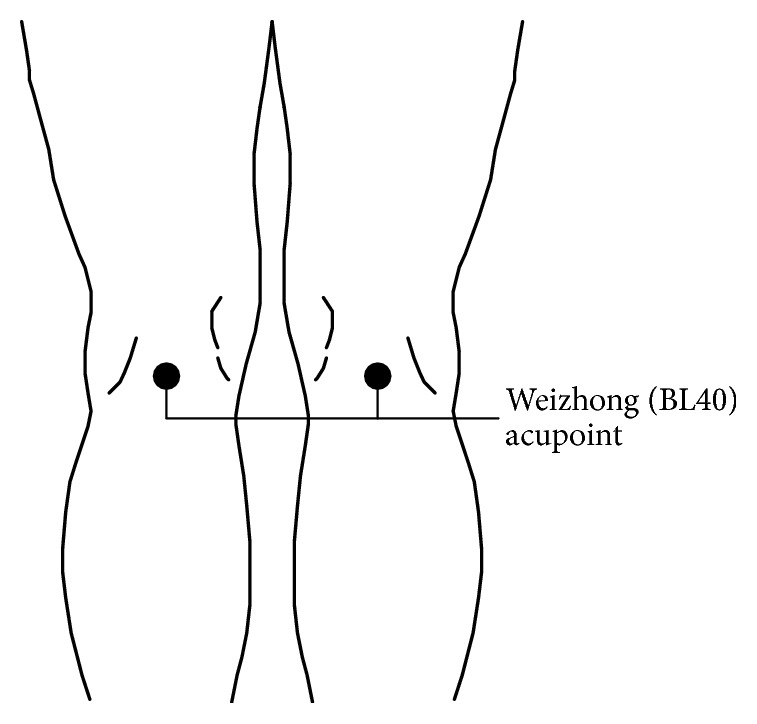
Weizhong (BL40) acupoint.

A 4-channel 808 nm LLLT instrument LA400 (United Integrated Services Co., Ltd., Taiwan; output power, 40 mW; frequency, 20 Hz; duty cycle, 50%; dosage, 15 J/cm^2^) with near infrared light was used. Laser acupuncture was applied on the Weizhong (BL40) acupoints (Figure 1) of the popliteal fossa and the Ashi acupoints on the back muscles for 10 minutes. After laser acupuncture, four 6 cm DongBang cups (DongBang Acupuncture, Kyunggi-do, Korea) were used for Chinese cupping. After the cups were placed on the lower back muscles at the level of the L2–5 lumbar spinal disks, suction of each cup was applied until 1 cm of the skin was drawn up and then held for 5 minutes. The same treatment protocol was followed for the control group; however, patients in this group received sham LLLT (laser instrument number 2) during laser acupuncture. The physician (Lin M. L.) administered the treatment to all patients between 3 and 6 pm (exuberant flowing time of the bladder meridian) for 5 continuous days.

#### 2.3.1. Acupoint Selection

The patients were positioned on a treatment table with their knees maintained in slight flexion. The Weizhong (BL40) acupoint was palpated at the midpoint of the transverse crease and located between the biceps femoris and semitendinosus tendons on the popliteal fossa. Subsequently, the lower back muscles of the patients were palpated, and the tender point was then considered the Ashi acupoint. The acupoints were selected according to the acupoint theory of the traditional Chinese medicine and were treated by the physician (Lin M. L.).

### 2.4. Outcome Measures

#### 2.4.1. Pain

The VAS scores were recorded to assess the intensity of LBP at baseline and during days 1–5 of the treatment session. After the treatment session, the patients were allowed to rest for approximately 15 minutes, and their VAS scores were subsequently recorded. The tender points of their lower back muscles were palpated, and pain intensity of tenderness was recorded by drawing a marker on a 100-mm VAS. The same assessor performed the VAS assessment and was blinded to the group assignment.

#### 2.4.2. Blood Test

Plasma cortisol levels were analyzed before and after the treatment session. Blood samples were obtained to measure cortisol levels before and after the treatment session. Two 5-mL blood samples were obtained two times from the cubital vein of each patient. These blood samples were allowed to stand for 30 minutes, followed by sample centrifugation to obtain the plasma samples. Finally, the blood supernatants were collected in two 1-mL tubes. All plasma cortisol samples were frozen at −70°C. One blood sample was analyzed before the treatment on day 1, and the other sample was obtained after treatment on day 5. The cortisol analysis was performed at the Taipei Municipal Chung-Hsin Hospital.

### 2.5. Statistical Analyses

Data of the patients were analyzed by using the SPSS statistical software (SPSS Version 17.0, Chicago, Illinois, USA). The per-protocol analysis was used to analyze the outcomes of laser acupuncture group and control group. Comparing between two groups, the demographic data, VAS, and cortisol level were analyzed using Mann–Whitney *U* test and chi-square test. The VAS scores and plasma cortisol levels before and after the treatment were compared using the paired-sample *t*-test. The differences between the two groups were analyzed using the independent *t*-test. All statistical tests were two-tailed, and *p* < 0.05 was considered statistically significant.

## 3. Results

**Figure 2:**
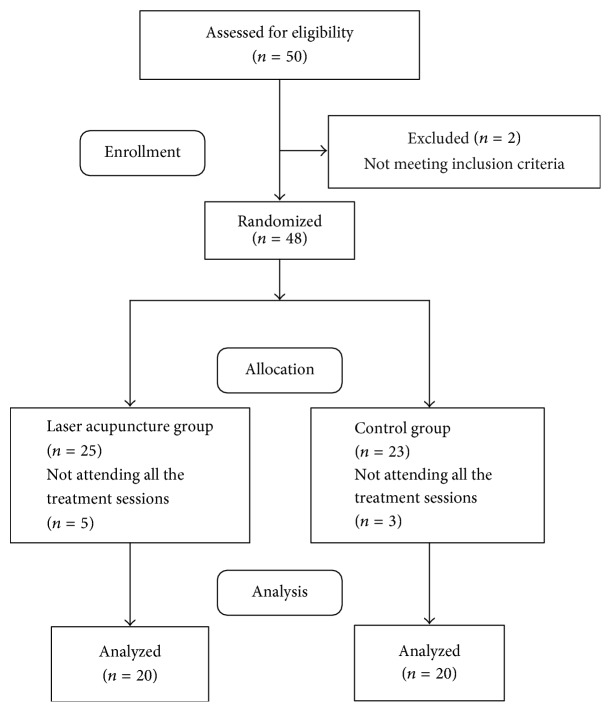
Consort flow diagram.

In this study, 48 patients with chronic nonspecific LBP participated, and 40 patients completed the study procedure (Figure 2). Of the 25 patients in the laser acupuncture group, 20 patients completed the treatment and 5 patients dropped out. Of the 23 patients in the control group, 20 patients completed the treatment and 3 patients dropped out. In two groups, the same reason of dropping out was that the patients were too busy to complete the treatment session. There were no adverse events in the both groups. Significant differences were not observed in the age, weight, height, and body mass index of the two groups in per-protocol analysis (*p* > 0.05, Table 1).

The baseline plasma cortisol levels did not significantly differ between the laser acupuncture and control groups (*p* = 0.65), and plasma cortisol levels for each group were no significantly different after the treatment (*p* = 0.93; Table 2). A comparison between the cortisol levels in the laser acupuncture group before and after the treatment demonstrated increased plasma cortisol levels in 6 patients, significantly decreased levels in 13 patients, and unchanged levels in 1 patient. In the control group, 6 and 14 patients showed increased and decreased cortisol levels, respectively. Moreover, plasma cortisol levels did not significantly decrease in both groups, and the effect size was 0.14 (95% CI = −0.50~0.78).

**Figure 3:**
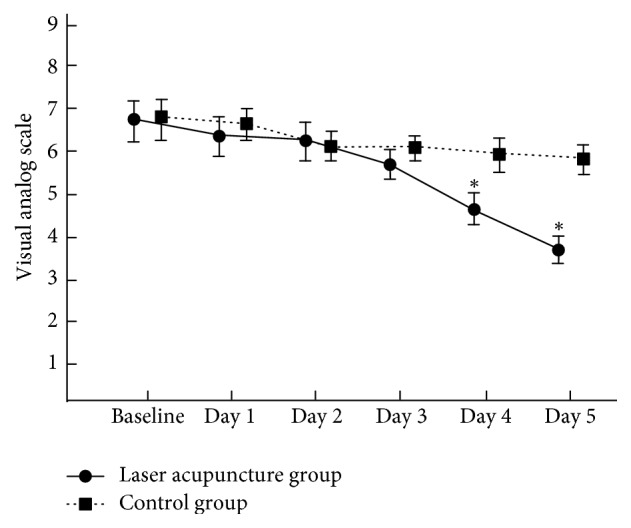
The change of VAS (mean and 95% confidence intervals) in two groups during the treatment session. ^*∗*^*p* < 0.05.

There was no significant difference in VAS between groups before the treatment (*p* = 0.44), but the VAS score was significantly lower in the laser acupuncture group than in the control group after the treatment (*p* = 0.005). The VAS scores of all patients before and after the treatment were significantly decreased in both groups (Table 2), and the effect size was −0.94 (95% CI = −1.62~−0.27). Compared with the control group, the laser acupuncture group exhibited significantly decreased VAS scores at days 4 and 5 (*p* < 0.05; Figure 3). Furthermore, the laser acupuncture group tended to have lower VAS scores than the control group.

## 4. Discussion

LBP is the most common disease worldwide, and total treatment costs for LBP exceed $100 billion per year in the United States [15]. Medical expenditure of patients with LBP has increased substantially. Therefore, we explored the laser acupuncture plus Chinese cupping to alleviate pain and plasma cortisol levels in patients with chronic nonspecific LBP. The Weizhong (BL40) acupoint is an acupuncture point of the bladder meridian with two branches traveling down from the upper back to the sacrum near the spine. The qi and blood circulation of the bladder meridian converge on the Weizhong (BL40) acupoint. According to traditional Chinese medicine, qi and blood stagnation leading to yang deficiency are the causes of LBP. Liu et al. indicated that massage, Chinese cupping, and acupuncture on the Weizhong (BL40) acupoint can improve qi and blood circulation and reduce LBP [4]. Our findings are consistent with the results of previous studies [4, 13], which showed that laser acupuncture plus Chinese cupping alleviates chronic nonspecific LBP.

A pulse semiconductor laser (wavelength, 808 nm; output power, 40 mW; energy density, 15 J/cm^2^) was used in this study. Of the 50 patients with chronic nonspecific LBP recruited in this study, 40 completed the treatment. The plasma cortisol levels of each patient were recorded before and after the treatment. Furthermore, all patients received equal attention; low dose acetaminophen was administered to the laser acupuncture and control groups during the study. Moreover, the patients were allowed to withdraw from the study if they did not want to follow the treatment protocol. The results showed most significant changes in the cortisol levels and VAS scores of the two groups after the treatment, with significant changes on days 4 and 5. This study demonstrated significantly decreased plasma cortisol levels in the two groups. The plasma cortisol levels were more affected in the laser acupuncture group than in the control group. Although the plasma cortisol levels were significantly decreased in the two groups, some patients had increased plasma cortisol levels. In both groups, increased plasma cortisol levels of some patients after the 5-day treatment might indicate that the accumulative dosage was inadequate and could not reduce their plasma cortisol levels. The previous study showed that serum cortisol levels increased significantly after needling on traditional acupuncture loci [16]. Furthermore, in our study, Chinese cupping reduced the plasma cortisol levels of the control group despite the inactive laser. Kim et al. suggested that compared with usual care (*p* < 0.01) and analgesia (*p* < 0.001), Chinese cupping significantly reduced LBP in randomized clinical trials [17]. Figure 2 indicates that the plasma cortisol levels were more decreased in the laser acupuncture group than in the control group. According to our study data, patients with chronic nonspecific LBP experienced pain relief on days 4 and 5.

Laser beams are monochromatic, coherent, and collimated. Energy power, wavelength, and energy density are the three important parameters of laser acupuncture. Energy power of the LLLT was considered as the treatment dose [13]. A systematic review by Baxter et al. demonstrated the moderate effects of laser acupuncture (energy power, 10 mW) in reducing muscular pain [18]. Ceylan et al. applied laser acupuncture with 8 mW of energy power on myofascial pain, which had an effect size of 0.81 for pain relief [19]. Laser acupuncture with 11.20 mW of energy power has been used for alleviating myofascial pain and fibromyalgia, with effect sizes of 0.97 and 1.31, respectively, for pain relief [20, 21]. We used laser acupuncture with 40 mW of energy power to decrease the VAS scores of LBP with an effect size of 1.21. The analgesic effects of laser acupuncture with adequate energy power were confirmed.

The wavelength and energy density of laser acupuncture are related to the energy absorbed from the laser radiation by superficial tissues [21]. A previous study revealed that maximum penetration of the deeper tissue was achieved at 770–850 nm wavelength of the LLLT [22]. Hudson et al. measured the energy density across different thicknesses of the sample tissue and revealed that 808 nm wavelength of the LLLT had improved penetration [23]. Anders and Wu indicated that the depth of penetration is a crucial factor and reported that a pulsed laser output can achieve improved penetration effects compared with a continuous wave laser output [24]. Lin et al. applied laser acupuncture with a pulsed laser output and 15 J/cm^2^ of energy density in patients with LBP and demonstrated that suitable parameters can be used on the acupoint [14]. Therefore, the parameters (wavelength, 808 nm; energy density, 15 J/cm^2^) of the pulsed laser output could be used for laser acupuncture treatment and to exert positive effects following the Arndt–Schulz law [12].

Laser acupuncture is a new noninvasive treatment and is easy to use. Compared with needle acupuncture, laser acupuncture is advantageous because of decreased instances of infection and lack of pain from needles [18]. According to traditional Chinese medicine, qi is a type of body energy that can be obtained through receiving needle acupuncture treatment [3]. Laser acupuncture does not provide physical stimulation to obtain qi but exerts photostimulation effects through LLLT to cause biomodulation. The physiological mechanism of LLLT has been adequately established in cellular experiments. LLLT has been used in culture experiments, in which it affects the survival of plasmids in* Escherichia coli* cells, and DNA repair was produced by pulsed emission modes through low-level infrared laser action [25]. Furthermore, AlGhamdi et al. demonstrated that LLLT is useful in enhancing the proliferation rate of cell lines [26]. LLLT alters photoreceptor functions and subsequent cellular signaling and cellular functions [27]. Five hypotheses describe the primary reactions after laser light absorption, namely, the single-oxygen hypothesis, redox property alteration hypothesis, nitric oxide hypothesis, transient local heating hypothesis, and superoxide anion hypothesis [28]. Secondary reactions of photobiomodulation involved a cellular signaling pathway, which involves the cell between the photoacceptor and a nucleus [28, 29]. The chemical energy within the cell can be converted in the form of adenosine triphosphate, which leads to pain relief and cell proliferation after the absorption of photonic energy. The tissues on acupoints were excited through LLLT, thus obtaining qi, similar to the process involved in the needle acupuncture technique [12]. Therefore, laser acupuncture can exert analgesia effects in accordance with traditional Chinese medicine acupuncture theory.

The hypothalamopituitary-adrenal axis is involved in stressors that play a role in chronic pain [30]. It is the principal neuroendocrine system that activates the central nervous system of mammals and produces cortisol as end product of being affected by stressors. The present results revealed that pain is alleviated by decreased stress hormone (i.e., cortisol) levels after a 5-day treatment session. The pain was significantly reduced as depicted by the VAS scores after laser acupuncture plus Chinese cupping. However, the changes in pain severity were not apparent when patients received only Chinese cupping five times. The patients with chronic nonspecific LBP experienced pain relief after five treatment sessions. Therefore, we proposed that the improvements after laser acupuncture and Chinese cupping at the Weizhong (BL40) acupoints are similar to the effects exerted by traditional acupuncture. In Taylor's study, cortisol levels decreased when *β*-endorphin was below the basal level in human participants [31]. In future studies, we suggested using an enzyme-linked immunosorbent assay to evaluate the variations in *β*-endorphin and cortisol levels in patients with chronic nonspecific LBP. It is an in-depth exploration where laser acupuncture plus Chinese cupping for consecutive periods may alleviate LBP and influence the relative *β*-endorphin and cortisol levels.

There are several limitations in this study. First, VAS scale is a clinical tool for self-perception pain assessment. However, usage of pain pressure algometer can be advantageous for quantifying the pain threshold on affected muscle in LBP patients. Second, LBP could affect the patients' activities of daily living and functional ability, so comprehensive evaluations for LBP patients, that is, assessments of disability or functional limitation, could get more outcomes of the treatment. Third, our study also lacks follow-up, and it cannot prove the long-term effects of laser acupuncture plus Chinese cupping.

## 5. Conclusion

The present results indicated that laser acupuncture combined with Chinese cupping at the Weizhong (BL40) and Ashi acupoints can alleviate LBP symptoms. The variations in plasma cortisol levels indicated that laser acupuncture plus Chinese cupping is an effective pain relief treatment. Moreover, laser acupuncture plus Chinese cupping at the Weizhong (BL40) and Ashi acupoints effectively reduces LBP. This therapy could be a suitable treatment option for patients with chronic nonspecific LBP in clinical settings.

## Figures and Tables

**Table 1:** Demographic data of two groups.

	Laser acupuncture group (*n* = 20)	Control group (*n* = 20)	*p*
Sex (male/female)	5/15	6/14	0.50
Age (years)	63.09 ± 16.19	63.70 ± 15.69	0.76
Height (cm)	159.66 ± 9.10	158.60 ± 7.13	0.64
Weight (kg)	61.84 ± 10.69	54.30 ± 6.97	0.06
BMI (kg/m^2^)	24.26 ± 10.10	21.59 ± 7.05	0.78
Onset duration (years)	4.13 ± 3.59	3.98 ± 2.83	0.67

**Table 2:** Effects on VAS and cortisol level before and after the treatment session.

	Laser acupuncture group (*n* = 20)			Control group (*n* = 20)		
	Before	After	*p* ^a^	Before	After	*p* ^a^
VAS	6.75 ± 1.46	4.20 ± 1.88^*∗*^	0.001	6.84 ± 1.41	5.80 ± 1.41	0.001
Cortisol level	11.41 ± 4.57	8.90 ± 3.78	0.01	10.57 ± 4.74	8.38 ± 3.64	0.03

*p* ^a^ meant the significant difference before and after treatment session. ^*∗*^*p* < 0.05, laser acupuncture versus control group.
